# Preliminary study of the characteristics of rib fractures and their impact on pulmonary ventilatory function

**DOI:** 10.1186/s13019-021-01538-3

**Published:** 2021-05-31

**Authors:** Weiming Wu, Tiancheng Zhao, Yang Li, Xiang Guo, Weiwei He, Yi Yang

**Affiliations:** Department of Thoracic Surgery, Shanghai Jiao Tong University Affiliated Sixth People’s Hospital, No. 600 Yishan Road, Shanghai, 200233 China

**Keywords:** Pulmonary ventilation function, Rib fracture, Pain level, Vital capacity, Multiple fractures

## Abstract

**Background:**

This study aimed to investigate the pulmonary ventilation function (PVF) according to different types of rib fractures and pain levels.

**Methods:**

This was a retrospective study of patients with thoracic trauma admitted to our ward from May 1, 2015, to February 1, 2017. Vital capacity (VC), forced expiratory volume in 1 s (FEV1), and peak expiratory flow (PEF) were measured on admission. A numerical rating scale (NRS) was used for pain assessment.

**Results:**

A total of 118 (85 males and 33 females) were included. The location of rib fractures did not affect the PVF. When the number of rib fractures was ≥5, the PVF was lower than in those with ≤4 fractures (VC: 0.40 vs. 0.47, *P* = 0.009; FEV1: 0.37 vs. 0.44, *P* = 0.012; PEF: 0.17 vs. 0.20, *P* = 0.031). There were no difference in PVF values between rib fractures with multiple locations and those with non-multiple locations (VC: 0.41 vs. 0.43, *P* = 0.202; FEV1: 0.37 vs. 0.39, *P* = 0.692; PEF: 0.18 vs. 0.18, *P* = 0.684). When there were ≥ 5 breakpoints, the PVF parameters were lower than those with ≤4 breakpoints (VC: 0.40 vs. 0.50, *P* = 0.030; FEV1: 0.37 vs. 0.45, *P* = 0.022; PEF: 0.18 vs. 0.20, *P* = 0.013). When the NRS ≥ 7, the PVF values were lower than for those with NRS ≤ 6 (VC: 0.41 vs. 0.50, *P* = 0.003; FEV1: 0.37 vs. 0.47, *P* = 0.040; PEF: 0.18 vs. 0.20, *P* = 0.027).

**Conclusions:**

When the total number of fractured ribs is ≥5, there are ≥5 breakpoints, or NRS is ≥7, the VC, FEV1, and PEF are more affected.

**Trial registration:**

The trial was conducted in accordance with the Declaration of Helsinki (as revised in 2013). The study was approved by the Ethics Committee of Shanghai Jiao Tong University Affiliated Sixth People’s Hospital, and individual consent for this retrospectively registered analysis was waived.

## Background

Rib fractures are frequent in trauma victims, seen in up to 39% of patients following blunt chest trauma and present in 10% of all trauma admissions [1, 2]. The number of rib fractures is often related to the severity of thoracic trauma [1]. A previous study has shown that patients with rib fractures often have declined pulmonary ventilation function (PVF), improving with surgical fixation [3]. Multiple rib fracture patients show symptomatic improvement associated with pain, quality of life, mobility, disability, and lung function over 1-year post-surgery [4]. The better PVF requires the chest wall has a normal shape and full movement. The rib plays an important role in completing this process since the rib is the main supporting structure of the chest wall. Second, the movement of the rib along a certain direction under the action of intercostal muscles can produce changes in thoracic volume. The larger the movement of the ribs, the larger the change in thoracic volume. Therefore, greater changes in gas pressure inside the thorax will lead to larger amounts of gas being exchanged to achieve pulmonary ventilation [5, 6]. Therefore, once rib fractures happen, it will cause some degree of decline in pulmonary ventilation function.

A previous study showed that declined PVF in patients with rib fractures likely to have respiratory complications such as pulmonary infection, atelectasis, and even respiratory failure [7]. Kanta S et al. stated that the risk of death rises in people with rates of decline of PVF exceeding around 60 ml/year, which is statistically significant with declines of 90 ml/year or beyond [8]. Another previous study indicated that if the PVF declines to a certain extent, and the patients are more likely to be admitted to the intensive care unit [9]. A study predicted acute respiratory failure (ARF) in patients with rib fractures and found that the number of rib fractures, tube thoracostomy, any lower-third rib fracture indicated a higher risk of ARF [10]. Nevertheless, those studies did not examine whether the kind of rib fracture, just as the site of rib fracture on the chest wall and the multiple locations or not and the number of segment of rib fracture will affect PVF or not. Besides, how the degree of pain affects pulmonary ventilation function as a significant and prominent subjective feeling in patients with rib fracture patients is mostly unknown. Therefore, the present preliminary study aimed to examine the factors that affect PVF in patients with rib fractures. We hope to provide some data about the decline in PVF that will be helpful to physicians for the management of rib fracture patients to avoiding ARF.

## Methods

### Study design and patients

This was a retrospective study of patients with thoracic trauma admitted to our ward from May 1, 2015, to February 1, 2017. All patients were tested for pulmonary ventilation function on admission.

The inclusion criteria were: 1) rib fracture without other injuries; 2) the time from injury was < 7 days; and 3) injury severity score (ISS) < 15. The exclusion criteria were: 1) smoking history; 2) chronic pulmonary disease; 3) received treatment before admission; 4) flail chest; 5) apparent pulmonary contusion (CT showed contusion with a total area of no more than one lobe of lung tissue); 6) pleural effusion (estimated total volume of effusion by ultrasonography > 500 mL); or 7) pneumothorax (CT manifestations of pulmonary compression volume > 30%).

### Examinations

Because the pulmonary ventilation function is affected by age and height, we first used the predictive equation [11] to calculate the predicted value of lung function for each patient. Then, the ratios of the values measured by the bedside portable pulmonary function detector and the predicted values calculated by the formula were calculated: ratio = measures value/predicted value. The ratio value was used as observation indexes for subsequent statistical analysis. The measurements of pulmonary ventilation function included vital capacity (VC), forced expiratory volume in 1 s (FEV1), and peak expiratory flow (PEF). All measurements were taken routinely by a respiratory specialist using a portable bedside pulmonary function detector. Each index was tested three times, and the mean values were adopted in the ratio calculated. The degree of pain was assessed using a numerical rating scale (NRS). First, the meaning of the number is explained to the patient (0: no pain; 1–3: mild pain; 4–7: moderate pain; 8–10: severe pain). Then, according to the degree of pain, the patient selects a number to represent his/her degree of pain. All measurements were completed on admission.

### Management

After the measurements were completed, the patients were given the necessary treatment such as analgesia, expectoration, and aerosol inhalation. The patients were treated surgically according to the indications for internal fixation [12].

### Date collection

The characteristics of the rib fracture site was determined according to 1) side: left, right, and bilateral; 2) anterior/lateral/posterior: anterior rib (from the parasternal line to the anterior axillary line), lateral rib (from the anterior axillary line to the posterior axillary line), and posterior rib (from the posterior axillary line to the paraspinal line); 3) location: high ribs (the first to fourth ribs), middle ribs (the fifth to eighth ribs), and low ribs (the ninth to twelfth ribs); 4) the total number of fractured ribs; 5) the total number of breakpoints (it refers to the place where the rib are broken); 6) fractures with multiple locations (two or more fractures on the one rib), and 7) pain.

### Statistical analysis

All data were analyzed using SPSS 18.0 (IBM, Armonk, NY, USA). The categorical data were displayed as n (%) and analyzed using the chi-square test or Fisher’s exact test. Continuous data were tested for normal distribution using the Kolmogorov-Smirnov test. Normally distributed data were presented as means ± standard deviation and analyzed using Student’s t-test. Otherwise, they were presented as medians (ranges) and analyzed using the Mann-Whitney U-test and Kruskal-Wallis test. Two-sided (except for the chi-square test) *P*-values < 0.05 were considered statistically significant.

## Results

### Characteristics of the patients

During the study period, 425 patients were admitted for trauma, but 118 (85 males and 33 females) could be included based on the inclusion/exclusion criteria of rib fracture patients, accounting for 27.8% of all traumatic patients during this period. Their characteristics are shown in Table 1.

**Table 1:** Characteristics of the patients with rib fractures

Characteristics	Values
n	118
Sex	
Male	85 (72.0%)
Female	33 (28.0%)
Age (years)	21–77 (53.4 ± 11.8)
Height (cm)	150–187 (167.7 ± 7.0)
Weight (kg)	44–105 (66.9 ± 11.3)
Body surface area (m^2^)	1.51–2.39 (1.84 ± 0.17)
Injury time (days)	0–7
Total number of fractured ribs	766
VC	0.43 (0.24–0.83)
FEV1	0.39 (0.20–0.77)
PEF	0.18 (0.07–0.70)

*VC* vital capacity, *FEV1* forced expiratory volume in one second, *PEF* peak expiratory flow

### Comparison of PVF in patients with rib fracture with different characteristics

There were no differences in PVF values according to the different side of rib fractures (Table 2). Rib fractures with multiple locations were not included in this analysis.

**Table 2:** Comparison of the pulmonary ventilation function of different sites of fracture on the chest wall

site	n	VC	*P*	FEV1	*P*	PEF	*P*
Left	60	0.43 (0.24–0.69)	0.292	0.38 (0.20–0.72)	0.627	0.18 (0.09–0.41)	0.567
Right	46	0.44 (0.28–0.83)		0.36 (0.24–0.66)		0.19 (0.07–0.70)	
Bilateral	12	0.36 (0.24–0.66)		0.35 (0.20–0.70)		0.19 (0.12–0.43)	
High	5	0.47 (0.32–0.54)	0.766	0.33 (0.30–0.56)	0.427	0.20 (0.15–0.32)	0.899
Middle	9	0.53 (0.27–0.83)		0.51 (0.26–0.77)		0.22 (0.15–0.41)	
Low	8	0.55 (0.50–0.60)		0.55 (0.42–0.68)		0.43 (0.16–0.70)	
Anterior	13	0.43 (0.32–0.83)	0.508	0.35 (0.27–0.77)	0.442	0.19 (0.13–0.34)	0.695
Lateral	19	0.45 (0.27–0.66)		0.41 (0.25–0.72)		0.19 (0.13–0.43)	
Posterior	12	0.48 (0.38–0.61)		0.43 (0.36–0.68)		0.18 (0.15–0.70)	

*VC* vital capacity, *FEV1* forced expiratory volume in one second, *PEF* peak expiratory flow

There were no difference in PVF values when separating the patients according to The number of rib fractures ≥4 vs. the number of rib fractures ≤3 (VC: 0.42 vs. 0.50, *P* = 1.137; FEV1: 0.38 vs. 0.42, *P* = 0.450; PEF: 0.18 vs. 0.20, *P* = 0.163), but when The number of rib fractures ≥5 vs. the number of rib fractures ≤4, significant differences were seen (VC: 0.40 vs. 0.47, *P* = 0.009; FEV1: 0.37 vs. 0.44, *P* = 0.012; PEF: 0.17 vs. 0.20, *P* = 0.031) (Table 3).

**Table 3:** Comparison of pulmonary ventilation function according to the total number of fractured ribs

Total	n	VC	*P*	FEV1	*P*	PEF	*P*
≤ 3	9	0.50 (0.27–0.64)	1.137	0.42 (0.26–0.68)	0.450	0.20 (0.15–0.70)	0.163
≥ 4	109	0.42 (0.24–0.83)		0.38 (0.20–0.77)		0.18 (0.07–0.45)	
≤ 4	25	0.47 (0.27–0.83)	0.009	0.44 (0.25–0.77)	0.012	0.20 (0.12–0.70)	0.031
≥ 5	93	0.40 (0.24–0.69)		0.37 (0.20–0.72)		0.17 (0.07–0.43)	

*VC* vital capacity, *FEV1* forced expiratory volume in one second, *PEF* peak expiratory flow

There were no significant differences in PVF values between rib fractures with multiple locations and those with non-multiple locations (VC: 0.41 vs. 0.43, *P* = 0.202; FEV1: 0.37 vs. 0.39, *P* = 0.692; PEF: 0.18 vs. 0.18, *P* = 0.684) (Table 4).

**Table 4:** Comparison of the pulmonary ventilation function according to multiple fracture or not

Multiple fractures	n	VC	*P*	FEV1	*P*	PEF	*P*
No	55	0.43 (0.24–0.83)	0.202	0.39 (0.20–0.77)	0.692	0.18 (0.07–0.43)	0.684
Yes	63	0.41 (0.24–0.67)		0.37 (0.20–0.68)		0.18 (0.09–0.70)	

*VC* vital capacity, *FEV1* forced expiratory volume in one second, *PEF* peak expiratory flow

There were no differences in PVF values when considering the number of breakpoints ≤3 vs. the number of breakpoints ≥4 (VC: 0.42 vs. 0.47, *P* = 0.303; FEV1: 0.37 vs. 0.38, *P* = 0.754; PEF: 0.18 vs. 0.20 *P* = 0.243), but for patients with ≥5 breakpoints, the PVF was lower than that of the patients with ≤4 breakpoints (VC: 0.40 vs. 0.50, *P* = 0.030; FEV1: 0.37 vs. 0.45, *P* = 0.022; PEF: 0.18 vs. 0.20 *P* = 0.013) (Table 5).

**Table 5:** Comparison of the pulmonary ventilation function according to the breakpoint

Breakpoint	n	VC	*P*	FEV1	*P*	PEF	*P*
≤ 3	7	0.47 (0.27–0.83)	0.303	0.38 (0.26–0.77)	0.754	0.20 (0.15–0.34)	0.243
≥ 4	111	0.42 (0.24–0.69)		0.37 (0.20–0.72)		0.18 (0.07–0.70)	
≤ 4	18	0.50 (0.27–0.83)	0.030	0.45 (0.26–0.77)	0.022	0.20 (0.13–0.70)	0.013
≥ 5	100	0.40 (0.24–0.69)		0.37 (0.20–0.72)		0.18 (0.07–0.43)	

*VC* vital capacity, *FEV1* forced expiratory volume in one second, *PEF* peak expiratory flow

There were no differences in PFV values when considering NRS ≤ 3 vs. ≥ 4 (VC: 0.67 vs. 0.42, *P* = 0.061; FEV1: 0.62 vs. 0.38, *P* = 0.550; PEF: 0.29 vs. 0.18, *P* = 0.053) or NRS ≤ 4 vs. ≥ 5 (VC: 0.48 vs. 0.42, *P* = 0.258; FEV1: 0.45 vs. 0.38, *P* = 0.096; PEF: 0.24 vs. 0.18, *P* = 0.082). The VC and FEV1 were lower in patients with NRS ≥ 6 compared with those with NRS ≤ 5 (VC: 0.41 vs. 0.50, *P* = 0.048; FEV1: 0.38 vs. 0.47, *P* = 0.040; PEF: 0.18 vs. 0.20, *P* = 0.134). All three index of PVF were lower in patient with NRS ≥ 7 compared with those patients NRS ≤ 6 (VC: 0.41 vs. 0.50, *P* = 0.048; FEV1: 0.38 vs. 0.47, *P* = 0.040; PEF: 0.18 vs. 0.20, *P* = 0.027) (Table 6).

**Table 6:** Comparison of the pulmonary ventilation function according to the pain level (NRS)

NRS	n	VC	*P*	FEV1	*P*	PEF	*P*
≤ 3	2	0.67 (0.50–0.83)	0.061	0.62 (0.47–0.72)	0.550	0.29 (0.27–0.30)	0.053
≥ 4	116	0.42 (0.24–0.69)		0.38 (0.20–0.72)		0.18 (0.07–0.70)	
≤ 4	4	0.48 (0.34–0.83)	0.258	0.45 (0.37–0.77)	0.096	0.24 (0.19–0.30)	0.082
≥ 5	114	0.42 (0.24–0.69)		0.38 (0.20–0.72)		0.18 (0.07–0.70)	
≤ 5	7	0.50 (0.34–0.83)	0.048	0.47 (0.37–0.77)	0.040	0.20 (0.15–0.30)	0.134
≥ 6	111	0.41 (0.24–0.69)		0.38 (0.20–0.72)		0.18 (0.07–0.70)	
≤ 6	11	0.50 (0.34–0.83)	0.003	0.47 (0.37–0.77)	0.004	0.20 (0.15–0.34)	0.027
≥ 7	107	0.41 (0.24–0.68)		0.37 (0.20–0.72)		0.18 (0.07–0.70)	

*VC* vital capacity, *FEV1* forced expiratory volume in one second, *PEF* peak expiratory flow

## Discussion

This study aimed to investigate the PVF according to different types of rib fractures and pain levels. The results strongly suggest that when the total number of fractured ribs is ≥5, there are ≥5 breakpoints, or NRS is ≥7, the VC, FEV1, and PEF are more affected. Nevertheless, the patients with rib fractures will present a wide variety of conditions. Identifying those at higher risk of altered PVF could be useful for triage.

There are many indicators to measure pulmonary ventilation function. Here, VC and FEV1 were selected because they are routinely measured in patients with rib fractures at our hospital and because of their operability and accuracy [13]. First, we analyzed the PVF among patients with different locations of a single rib fracture. These locations (left vs. right vs. bilateral, anterior vs. posterior vs. lateral, and high vs. middle vs. low) did not affect the PVF parameters. Therefore, the location of a single rib fracture does not affect the PVF.

Rib fractures with multiple locations(two or more fractures on one rib) usually have a bigger impact to the PVF because it will lead to an inability of the chest wall to support the effective thoracic expansion and have been shown to lead to ARF [14]. Patients with severe trauma and flail chest will have a sharp decline in PVF followed by ARF occurs which required mechanical ventilation and increasing the risk of complications and hospitalization costs [15, 16], especially in elderly patients [7, 9, 10]. In the present preliminary study, there were no differences between patients with multiple locations and non-multiple locations rib fractures. This could be because patients with flail chest were excluded from the present study.

A previous study showed that higher numbers of fractured ribs would lead to a poorer prognosis in elderly patients [17]. In the present study, when comparing the PVF between patients with ≤4 vs. ≥ 5 fractured ribs, the latter was significantly lower in VC, FEV1, and PEF than the former group, suggesting that we should pay more attention to those patients with ≥5 fractured ribs. Then, we compared the effect of the total number of breakpoints (it refers to the place where the rib are broken). Similar results were observed, i.e., that the total number of breakpoints ≥5 were associated with a significant decline in PVF compared with the total number of breakpoints ≤4, suggesting that the number of breakpoints is possibly more clinically meaningful than the number of rib fractures. It is because the total number of rib fractures are not equal to the total number of breakpoints in sometimes.

Rib fractures can cause severe chest pain and affect the patients’ quality of life, especially in the early traumatic stage [18–20]. In the present study, PVF at admission, before any treatment, with cut-off points of ≥6 and ≥ 7 indicated worse PVF.

The clinical significance of rib fracture internal fixation for patients with flail chest has been confirmed [21]. However, the indications for patients without flail chest are still controversial [17, 22, 23]. Considering that the degree of decline in PVF is often closely related to adverse prognosis, the degree of change in PVF could be an indication for the internal fixation for rib fractures. Internal fixation surgery can reduce post-traumatic complications and promote the recovery of pulmonary function [24]. Nevertheless, this study was not designed to answer this question, and future studies will have to look into that.

This study has several limitations, which are as follows: (i) the number of patients was small. (ii) Only the data available in the patient charts could be analyzed. (iii) The treatment outcomes could not be examined because many patients are returned home and can go to other hospitals for follow-up. (iv) A previous study indicated that elderly patients are at increased risk of pulmonary complications. Because this study is a preliminary study on this topic, complications were not addressed in this current study, which should be addressed in future studies. (v) All the patients were treated surgically according to the indications for internal fixation following a study from a single institution, which may not be applicable to many other institutional practices. (vi) All of the 118 patients were treated with internal fixation for rib fractures, and there were no severe complications such as lung infections. Therefore, we could not test the association of the reduction in lung function with adverse complications such as pulmonary infections, ventilator-assisted ventilation, hospitalization, and ICU stays. (vii) The pain score was only performed on admission, and the impact of analgesia not taken into account when reporting PVFs.

## Conclusions

In conclusion, in patients with rib fractures, the VC, FEV1, and PEF are more affected when the total number of fractured ribs is ≥5, when there are ≥5 breakpoints, or when the NRS is ≥7.

## Data Availability

The datasets used and/or analyzed during the current study are available from the corresponding author on reasonable request.

## Abbreviations

PVF: Pulmonary ventilation function
ISS: Injury severity score
VC: Vital capacity
FEV1: Forced expiratory volume in 1 s
PEF: Peak expiratory flow
NRS: Numerical rating scale
ISV: Incentive spirometry volume
PEFR: Peak expiratory flow rate
ARF: Acute respiratory failure
